# Inhibitory Activities of Polyphenolic Extracts of Bangladeshi Vegetables against α-Amylase, α-Glucosidase, Pancreatic Lipase, Renin, and Angiotensin-Converting Enzyme

**DOI:** 10.3390/foods9070844

**Published:** 2020-06-29

**Authors:** Razia Sultana, Adeola M. Alashi, Khaleda Islam, Md Saifullah, C. Emdad Haque, Rotimi E. Aluko

**Affiliations:** 1 Department of Food and Human Nutritional Sciences, University of Manitoba, Winnipeg, MB R3T 2N2, Canada; sultana1@myumanitoba.ca (R.S.); monisola.alashi@umanitoba.ca (A.M.A.); 2 Institute of Nutrition and Food Sciences, University of Dhaka, Nilkhet Rd, Dhaka 1000, Bangladesh; arunadu15@gmail.com; 3 Natural Resources Management Division, Bangladesh Agricultural Research Council, Dhaka 1215, Bangladesh; m.saif@barc.gov.bd; 4 Natural Resources Institute, University of Manitoba, Winnipeg, MB R3T 2N2, Canada; CEmdad.Haque@umanitoba.ca; 5 The Richardson Centre for Functional Foods and Nutraceuticals, University of Manitoba, Winnipeg, MB R3T 2N2, Canada

**Keywords:** Bangladesh, vegetables, polyphenols, amylase, glucosidase, renin, angiotensin-converting enzyme, lipase, mass spectrometry

## Abstract

The aim of the study was to determine the in vitro enzyme inhibition activities of aqueous polyphenolic extracts of nine popular Bangladeshi vegetables, namely ash gourd, bitter gourd, brinjal, Indian spinach, kangkong, okra, ridge gourd, snake gourd, and stem amaranth. Polyphenolic glycosides were the major compounds present in the extracts. Inhibition of α-amylase (up to 100% at 1 mg/mL) was stronger than α-glucosidase inhibition (up to 70.78% at 10 mg/mL). The Indian spinach extract was the strongest inhibitor of pancreatic lipase activity (IC_50_ = 276.77 µg/mL), which was significantly better than that of orlistat (381.16 µg/mL), a drug. Ash gourd (76.51%), brinjal (72.48%), and snake gourd (66.82%) extracts were the most effective inhibitors of angiotensin-converting enzyme (ACE), an enzyme whose excessive activities have been associated with hypertension. Brinjal also had a significantly higher renin-inhibitory activity than the other vegetable extracts. We conclude that the vegetable extracts may have the ability to reduce enzyme activities that have been associated with hyperglycemia, hyperlipidemia, and hypertension.

## 1. Introduction

Several physiological disturbances can be attributed to the effect of metabolic syndrome, which is a condition characterized by disease conditions such as obesity, dyslipidemia, hyperglycemia, and hypertension [1]. These metabolic syndrome-related diseased are considered major health concerns worldwide. Obesity is considered as one of the main factors in the pathogenesis of cardiovascular diseases such as hypertension, stroke, type 2 diabetes mellitus (T2DM), and cancer [2,3]. Postprandial hyperglycemia (PPHG), a major feature of T2DM, occurs when the blood glucose level increases after consuming a meal and this is an important factor taken into consideration for diabetes management. PPHG can be controlled by inhibiting the enzymes responsible for causing elevated blood glucose [4].

Several epidemiological and empirical studies have reported that consumption of fruits and vegetables containing polyphenol compounds play an important role in inhibiting carbohydrate-hydrolyzing enzymes such as α-amylase and α-glucosidase [5]. α-amylase (EC 3.2.1.1) is responsible for the hydrolysis of α-1,4 glucosidic bonds of starches, which are then converted to oligosaccharides. These oligosaccharides are further converted to maltose, glucose, and limit dextrins, causing blood glucose levels to increase [6]. The enzyme α-glucosidase (EC 3.2.1.20), located in the brush border of the small intestine enterocytes, is responsible for carbohydrate breakdown and synthesis. α-glucosidase is a carbohydrate-hydrolase that releases monosaccharides, and is the main enzyme responsible for increasing blood glucose levels after consumption of a meal. Therefore, subsequent to the initial α-amylase hydrolysis of dietary carbohydrates, α-glucosidase plays an important role in ensuring the release of absorbable monosaccharides in the intestinal tract [7]. To control PPHG, acarbose, miglitol, and voglibose are drugs that are used as α-glucosidase and α-amylase inhibitors. However, these drugs not only are expensive but they also affect the gastrointestinal system negatively, with long-term use of these drugs causing flatulence and diarrhea [8].

Digestion of dietary triglycerides occurs in the small intestine by a key enzyme called pancreatic lipase (PL) to release 2-monoglyceride and fatty acids, which are then absorbed. Hence, inhibition of PL (EC 3.1.1.3) activity is regarded as a major approach for obesity prevention and treatment [9,10,11]. Although orlistat (a drug) has been used as a PL inhibitor, the negative side effects such as bloating, oily spotting, fecal urgency, steatorrhea, and fecal inconsistency have reduced acceptance [12,13].

Another reason for the development of cardiovascular diseases is hypertension, which causes arteriosclerosis, congestive heart failure, coronary heart disease, end-stage renal diseases, myocardial infarction, and stroke [14]. The renin–angiotensin system (RAS) has been found to be one of the major regulatory mechanisms in blood pressure regulation [15]. Within the RAS, renin (EC 3.4.23.15) acts on angiotensinogen to release angiotensin I, which is then cleaved by angiotensin-converting enzyme (ACE) to produce angiotensin II, a potent vasopressor. Therefore, ACE (EC 3.4.15.1) and/or renin inhibition help to manage hypertension and offer cardiovascular protection by limiting the physiological level of angiotensin II. Just like obesity and diabetes, current clinical management of hypertension involves mainly the use of drugs, which also have negative side effects [15].

Different in vitro and in vivo studies have observed that dietary phenolic compounds have many properties beneficial for maintaining human health [16]. In particular, studies have found that phenolic compounds are helpful for inhibiting lipase, alpha-amylase, alpha-glucosidase, ACE, and renin [17,18,19,20]. In fact, polyphenol-rich extracts have been shown to reduce blood pressure in spontaneously hypertensive rats and could serve as potential antihypertensive agents [21,22]. Therefore, this research investigated the enzyme inhibition ability of aqueous extracts of nine popular Bangladesh vegetables to determine their potential utility as agents against obesity (PL, α-glucosidase, α-amylase), diabetes (α-glucosidase, α-amylase), and hypertension (ACE, renin). Aqueous extracts were used in order to enhance the practical utilization of the research outcome, since the human gastrointestinal tract is highly hydrophilic and soluble compounds are more likely to be absorbed or interact with enzymes than hydrophobic compounds.

## 2. Materials and Methods

### 2.1. Materials

Fresh vegetables, namely ash gourd (BINA CHALKUMRA 1), bitter gourd (BARI KOROLA 1), brinjal (BARI BEGUN 8), Indian spinach (BARI PUISHAK 1), kangkong (BARI GIMAKOLMI 1), okra (BARI DHEROSH 2), ridge gourd (BARI JHINGA 1), snake gourd (BARI CHICHINGA 1), and stem amaranth (BARI DATA 1), were collected from the Bangladesh Agricultural Research Institute (BARI), Gazipur, Bangladesh. The vegetables were dried in the oven at 40 °C, ground into powders, and then stored at −20 °C. Porcine pancreatic α-amylase, porcine PL, rat intestinal acetone powder, acarbose, orlistat, 4-nitrophenyl α-D-glucopyranoside (PNP), N-(3-[2-furyl]acryloyl)-phenylalanyl glycyl glycine (FAPGG), and rabbit lung ACE were purchased from Sigma-Aldrich (St. Louis, MO, USA). A human recombinant renin inhibitor screening assay kit was purchased from Cayman Chemicals (Ann Arbor, MI, USA). Other analytical grade chemical reagents were obtained from Fisher Scientific Company (Oakville, ON, Canada).

### 2.2. Extraction of Polyphenolic Compounds

Water-soluble free (unbound) polyphenols were extracted using a previous method [23] with minor modifications as follows. Briefly, dried vegetable powders were weighed and one part mixed with 20 parts of distilled water (1:20) in a 500 mL beaker, which was then adjusted to 60 °C and stirred for 2 h [23]. After cooling to ambient temperature, the mixture was centrifuged for 30 min at 5600× *g*, and the supernatant passed through a muslin cloth to collect filtrate #1. The residue was mixed with water (1:20) and the extraction, centrifugation, and filtration processes repeated to collect filtrate #2, which was then combined with filtrate #1. The combined filtrate was partially evaporated in a rotary evaporator (Heidolph Instruments GmbH & CO., Schwabach, Germany) under vacuum (~24 mm Hg) at 60 °C, and the aqueous residue freeze-dried and stored at −20 °C.

### 2.3. UHPLC MS/MS Analysis of Polyphenolic Compounds

An Agilent 1290 UHPLC system (Santa Clara, CA, USA) coupled with an HSS T3 2.1 × 100 mm 1.7 µm column from Waters Corp (Milford, MA, USA) was used to perform UHPLC analysis of the vegetable extracts. Samples were mixed with distilled water, vortexed, and passed through a 0.2 µm filter. Then, a 5 µL portion of the filtrate was injected onto the column followed by elution at a flow rate of 0.5 mL/min using mobile phases A and B (0.1% formic acid in water and 0.1% formic acid in acetonitrile, respectively) at 40 °C. The following gradients were used: initial holding time 0.5 min, mobile phase B ramped up to 50% after 5 min, 95% after 6 min, held for 1 min, and re-equilibrated for 1.5 min. Compounds were identified using a diode array detector at a wavelength range of 230–640 nm in 2 nm increments and a frequency of 5 Hz. The mass spec was carried out in an Agilent 6550 QTOF (Santa Clara, CA, USA) at 200 °C, using a drying gas pressure of 18 psi, 40 psi nebulizer and 350 °C sheath gas, a pressure of 12 psi, and 3500 V capillary with a 1000 V nozzle, and ran in positive ion electrospray at a frequency of 3Hz and acquisition from 30–1700 *m/z*. The MS/MS was performed at a narrow quadruple setting (1.3 atomic mass units), using 10, 20, and 40 eV collision energy and 30–1700 *m/z*. The compounds were identified using MS/MS fragmentation patterns and quantified based on the MS peak area.

### 2.4. Inhibition of α-Amylase Activity

α-amylase inhibition assay was carried out using a previous method [14] with slight modifications. Plant extracts were dissolved in 1 mL of 0.2 mM sodium phosphate buffer, pH 6.9 containing 6 mM NaCl. Then, 100 µL of sample aliquot (0.03–10 mg/mL final concentration) and 100 µL of α-amylase solution were added together in a test tube and incubated for 10 min at 25 °C. A 100 µL amount of 1% starch (previously dissolved in the same buffer, heated, and cooled) was added to the mixture and incubated again at 25 °C for 10 min. Then, 200 µL of 96 mM dinitrosalicylic acid (DNSA), prepared in 2 M sodium potassium tartrate tetrahydrate, were added to terminate the reaction and heated in a water bath at 100 °C for 15 min. Subsequently, a 3 mL amount of double-distilled water was added after the reaction mixture was cooled down to room temperature. From this reaction mixture, a 200 µL aliquot was transferred to a 96-well microplate and absorbance read at 540 nm using a Synergy™ H4 microplate reader (Biotek™, Winooski, VT, USA) at 25 °C. The phosphate buffer was used as a blank and its absorbance subtracted from each well to calculate enzyme activity. Acarbose, a known α-amylase inhibitor, was used as standard and assayed concomitantly with the samples. The inhibitory activity of α-amylase was calculated using the following equation:

Inhibition (%)=(Ac−As/Ac)×100)

where Ac = Absorbance of the control (no inhibitor) and As = Absorbance of the sample.

### 2.5. Inhibition of α-Glucosidase Activity

α-glucosidase inhibitory activity of the samples was determined according to a previously described method [24] with the following modifications. First, a 300 mg portion of rat intestinal powder was mixed with 9 mL of 0.9% (*w*/*v*) NaCl solution and centrifuged at 5600× *g* for 30 min, and the supernatant was used as the source of α-glucosidase activity. Plant extracts were dissolved (final concentration of 0.03–10 mg/mL) in 0.1 M sodium phosphate buffer pH 6.9 and 50 µL mixed with 50 µL of the α-glucosidase solution in a 96-well microplate followed by incubation for 10 min at 37 °C. Then, 100 µL 5 mM (PNP) also dissolved in the phosphate buffer were added to each well and the absorbance read at 405 nm in 30 s intervals for 30 min using the Synergy™ H4 microplate reader with temperature maintained at 37 °C. A blank measurement was taken without the addition of the enzyme, and its absorbance was subtracted from each well. Acarbose, an α-glucosidase inhibitor, was used as standard and assayed using the same protocol. The following equation was used to determine the α-glucosidase inhibitory activity of the samples:

Inhibition (%)=(Ac−As)/(Ac)×100

where Ac = Absorbance of the control (no inhibitor) and As = Absorbance of the sample.

### 2.6. Inhibition of Lipase Activity

The method described by Tang et al. [25] with slight modifications was used to determine PL inhibition by measuring the release of 4-methyl umbelliferone (4MU) from 4-methyl umbelliferyl oleate (4MUO). PL solution (final concentration of 3.125 U/mL) was prepared in 13 mM Tris-HCl buffer, pH 8.0 containing 1.3 mM CaCl_2_ and 25 µL added to the mixture containing 225 µL of a 0.5 mM 4-MUO solution and 25 µL of sample (different concentrations) to start the enzyme reaction, followed by incubation for 1 h at 37 °C. The Synergy™ H4 microplate reader was set at 400 nm and used to measure the amount of 4MU released during the reaction. Orlistat, a commonly used pharmacological agent against PL, was used as the standard.

The following equation was used to calculate PL inhibition:

Inhibition (%)=(Ac−As)/(Ac)×100

where Ac = Absorbance of control (no inhibitor) and As = Absorbance of the sample.

### 2.7. ACE Inhibition Assay

The method described by Alashi et al. [21] was used to determine ACE-inhibitory activity of the polyphenolic extracts. ACE, FAPGG (ACE substrate), and samples were individually dissolved in 50 mM Tris-HCl buffer, pH 7.5 containing 0.3 M NaCl. A 10 µL aliquot of ACE (final reaction activity 25 mU) was added to each well containing 170 µL of 0.5 mM FAPPG and 20 µL of samples at 37 °C. The buffer was used as blank (uninhibited reaction), while captopril, an ACE-inhibitory drug, was used as standard and assayed using a similar protocol. Absorption was read at 345 nm at 1 min intervals for 30 min to determine the reaction rate. The slope of the blank or sample reactions was used to calculate the percentage ACE inhibition as follows:

$$\mathrm{ACE}\;\mathrm{inhibition}\;\left(\%\right)=\left(\frac{\mathrm{Slope}\;{\left(∆A/\min\right)}_{\mathrm{blank}}-\mathrm{Slope}\;{\left(∆A/\min\right)}_{\mathrm{sample}}}{\;\mathrm{Slope}\;(∆A/\min_{)\mathrm{blank}}}\right)\times 100)$$

### 2.8. Renin Inhibition Assay

The renin-inhibitory activity of samples was determined using a previously described method [26]. Briefly, 20 µL of the substrate, 160 µL of assay buffer, and 10 µL of distilled water were mixed and added to the background well. Then, 20 µL of the substrate, 150 µL of assay buffer, and 10 µL of water were mixed into the control (uninhibited) wells, whereas the sample (inhibited) wells contained same reagents except that the water was replaced with 10 µL of samples (0.5 mg/mL assay concentration). This was followed by the addition of 10 µL of renin solution (dissolved in the assay buffer) to the control and sample wells to initiate enzyme reaction; the microplate was shaken for 10 s to ensure adequate mixing of the reagents and then incubated at 37 °C for 10 min in the dark. Enzyme catalytic activity was measured as the fluorescence intensity (FI) measured at excitation and emission wavelengths of 340 and 490 nm, respectively. Enzyme inhibition was calculated as follows after subtracting the FI of the background well from the control and sample wells:

Renin inhibition (%)=(FI of control well−FI of sample well)/( FI of control well)×100

### 2.9. Statistical Analysis

A minimum of duplicate assays were used to find out the mean values and standard deviations. For statistical analysis, analysis of variance (Kruskal–Wallis ANOVA) was used, while significant differences (*p* < 0.05) between mean values were determined by the Duncan’s multiple range tests. The IBM SPSS statistical package (version 24, Armonk, NY, USA) was used for all statistical analyses.

## 3. Results

### 3.1. UHPLC MS/MS Analysis

The major polyphenolic compounds identified in the vegetable extracts are shown in Table 1. More polyphenolic compounds were detected from Indian spinach, kangkong, and okra as compared to other vegetable extracts. The identified compounds were mainly chlorogenic acid and the glycosides of quercetin, vitexin hexoside, and kaempferol. Kaempferol O-sophoroside was particularly present at a very high concentration in snake gourd and may be the main compound that determined the bioactive properties of this extract.

### 3.2. α-Amylase Inhibition

**Figure 1:**
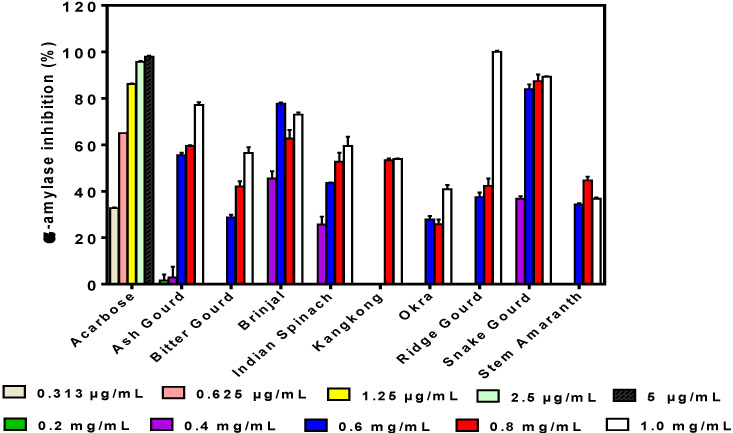
Inhibition of α-amylase activity at different concentrations of acarbose and aqueous polyphenolic extracts of vegetables. Bars are means (*n* = 3) ± standard deviation.

The inhibitory activity of α-amylase obtained in this study by the phenolic extracts was mostly dose-dependent, although at higher concentrations than acarbose, the standard compound (Figure 1). However, for brinjal and stem amaranth, decreases at sample concentrations >0.6 and 0.8 mg/mL, respectively, were observed. Only ash gourd had detectable activity at 0.2 mg/mL, whereas only brinjal, Indian spinach, and snake gourd had high inhibition levels at 0.4 mg/mL. Kangkong had no detectable activity at 0.2–0.6 mg/mL. Among the vegetable extracts, the ridge gourd achieved 100% inhibition at 1 mg/mL concentration, which is significantly (*p* < 0.05) better than the other extracts. The okra (40.82% ± 1.88%) and stem amaranth (36.84% ± 0.49%) showed the lowest levels of α-amylase inhibition, respectively, at 1 mg/mL.

### 3.3. α-Glucosidase Inhibition

**Figure 2:**
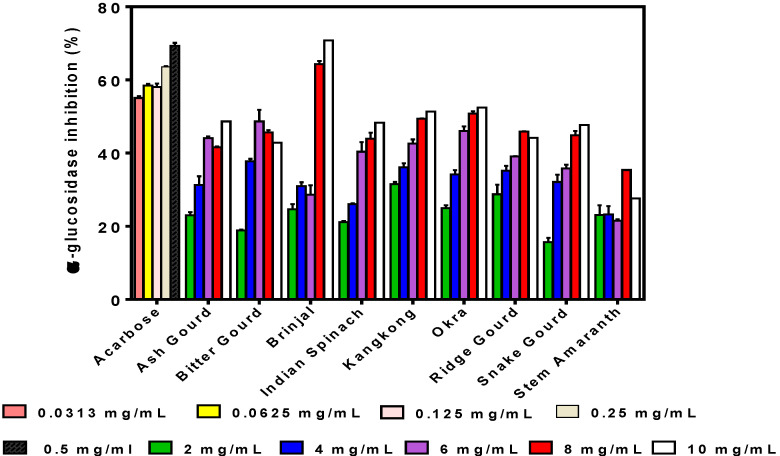
Inhibition of α-glucosidase activity at different concentrations of acarbose and aqueous polyphenolic extracts of vegetables. Bars are means (*n* = 3) ± standard deviation.

A mostly dose-dependent effect was also observed as the inhibitory activity of α-glucosidase increased with increasing concentration for almost all the samples (Figure 2). However, decreases in α-glucosidase inhibition were observed at sample concentrations >6 and 8 mg/mL for bitter gourd and stem amaranth, respectively. The highest inhibition was observed for brinjal (70.78% ± 3.45%) at 10 mg/mL, which is similar to that of acarbose (69.36% ± 0.80% at 0.5 mg/mL), a purified synthetic inhibitor of α-glucosidase. With the exception of brinjal (higher activity) and stem amaranth (lower activity), the inhibitory values obtained for the vegetable extracts were similar at 10 mg/mL, but snake gourd had the lowest inhibition at 0.2 mg/mL.

### 3.4. Pancreatic Lipase (PL)-Inhibitory Activity

**Figure 3:**
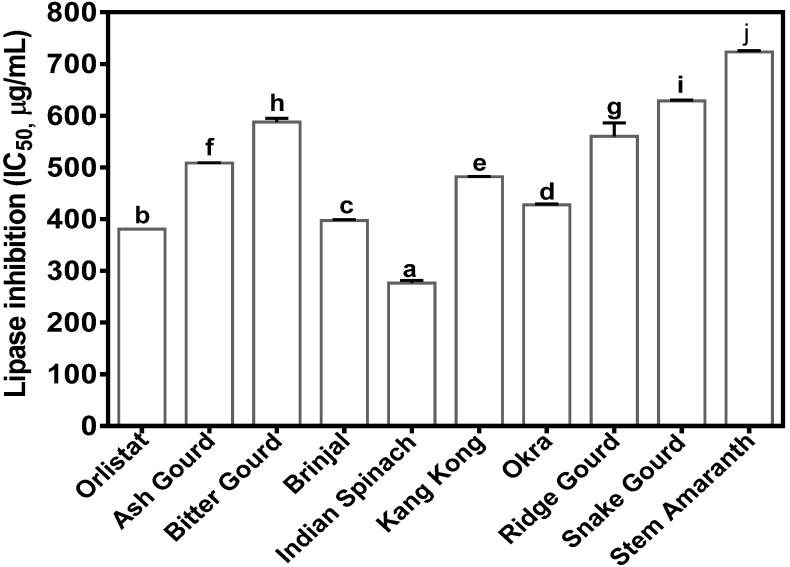
IC_50_ values (*n* = 3 ± standard deviation) for the inhibition of pancreatic lipase (PL) activity by orlistat and aqueous polyphenolic extracts of vegetables. Bars with different letters have mean values that are significantly (*p* < 0.05) different.

PL inhibition was strong for all the vegetable extracts, which enabled IC_50_ calculation as shown in Figure 3. Because lower IC_50_ values indicate stronger inhibitory activity, the results obtained show that Indian spinach (276.77 ± 4.95 µg/mL) was the most active with a significantly (*p* < 0.05) lower value than orlistat. Brinjal (397.22 ± 2.36 µg/mL), okra (427.94 ± 1.40 µg/mL), and kangkong (482.04 ± 0.67 µg/mL) also had strong activities, although lower than orlistat (381.16 µg/mL). It is also noticeable that the stem amaranth extract had the weakest PL inhibition (IC_50_ = 723.394 ± 2.36 µg/mL), just as it showed the lowest inhibitions of α-amylase and α-glucosidase inhibitory activities.

### 3.5. Angiotensin-Converting Enzyme (ACE)-Inhibitory Activity

**Figure 4:**
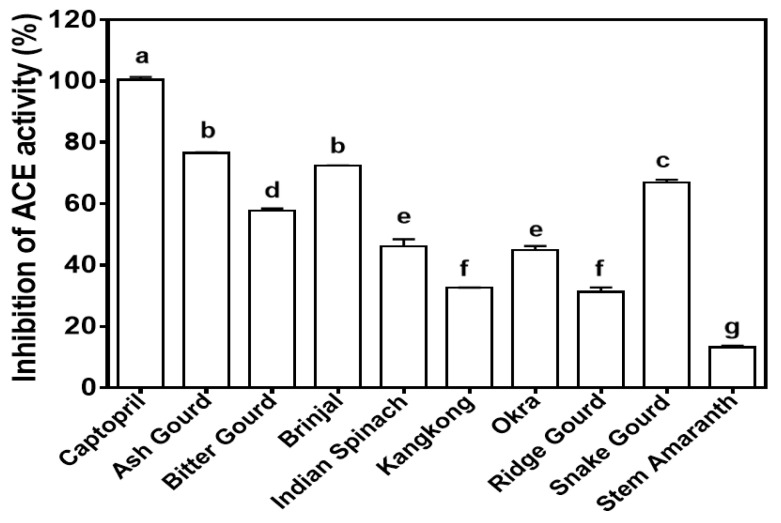
Inhibition of angiotensin-converting enzyme (ACE) activity (*n* = 3 ± standard deviation) by 1 mg/mL captopril and aqueous polyphenolic extracts of vegetables. Bars with different letters have mean values that are significantly (*p* < 0.05) different.

Different levels of ACE inhibition were obtained for the vegetable extracts but, in general, the ash gourd (76.51% ± 0.25%), brinjal (72.48% ± 0.02%), and snake gourd (66.82% ± 0.99%) were the most active (Figure 4). All the vegetable extracts had significantly (*p* < 0.05) weaker inhibition than captopril, the ACE-inhibitory drug. It is also observable that ash gourd also showed a good record for inhibiting α-amylase and α-glucosidase. Stem amaranth showed the lowest activity for all the concentrations and the lowest ACE inhibition, which is consistent with the observed poor inhibitory activities against PL, amylase, and glucosidase.

### 3.6. Renin-Inhibitory Activity

**Figure 5:**
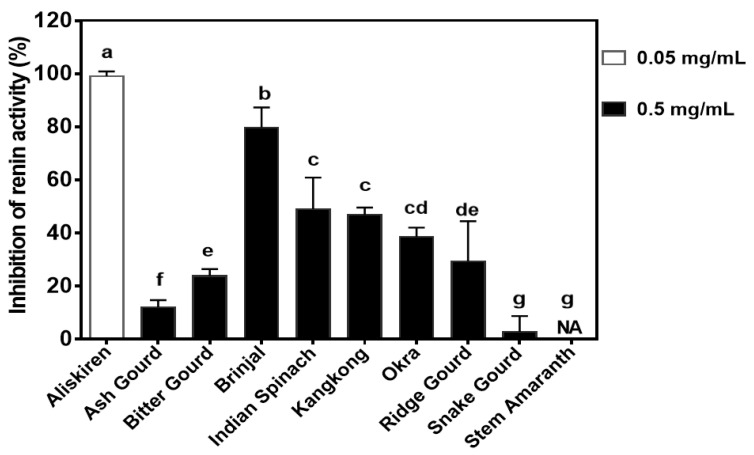
Inhibition of renin activity (*n* = 3 ± standard deviation) by aliskiren and aqueous polyphenolic extracts of vegetables. Bars with different letters have mean values that are significantly (*p* < 0.05) different.

The highest renin inhibitory activity was exhibited by brinjal (79.64% ± 7.63%) at 0.5 mg/mL as compared to 99.11% ± 1.75% for aliskiren (drug) at 0.05 mg/mL (Figure 5). The snake gourd had the significantly lowest renin inhibition, which was not significantly (*p* > 0.05) different from the stem amaranth with no detectable renin inhibition.

## 4. Discussion

UHPLC MS/MS analysis is considered an effective analysis method because it provides high selectivity, high sensitivity, and the potentiality for robust and accurate analysis identification of compounds [27]. As shown in Table 1, the dominant phenolic compounds were the glycosides, which reflect the aqueous extraction. The water solubility properties of the phenolic compounds could enhance their interactions with target enzymes within the hydrophilic environment of the gastrointestinal tract. For example, the aqueous extracts of some bean varieties were shown to have stronger lipase inhibition than the ethanolic extracts [28]. Inhibition of α-amylase was mainly dose-dependent except for brinjal and stem amaranth, where their decreases occurred at higher sample concentrations. However, the dose-dependent inhibitory activity pattern observed for most of the samples was also reported for *α*-amylase inhibition by bitter gourd extract [29]. The decreased α-amylase inhibition at high concentrations of brinjal and stem amaranth may be due to increased interactions between the polyphenol molecules, which reduced interactions with the enzyme protein. The weaker α-amylase inhibitory activity when compared to acarbose, which is an approved drug, suggests lower enzyme binding intensity by the polyphenolic compounds. A previous study also reported that the ethanolic extract of bitter gourd exhibited lower α-amylase activity than acarbose [29]. The high inhibitory activity of snake gourd (89.26% ± 0.23%) may be attributed to the kaempferol O-sophoroside, although the contribution of other non-identified compounds cannot be discounted. The low inhibitory activity of okra suggests that the quercetin glycosides, which were the main identified compounds in the extract, are not very effective inhibitors of α-amylase. In contrast, other extracts that contained vitexin, chlorogenic acid, and dicaffeoyl quinic acid had higher inhibitions of α-amylase activity when compared to okra that contained mainly quercetin glycosides. α-amylase inhibitors are also referred to as starch blockers because they prevent starch absorption. Digestive amylase enzyme and other secondary enzymes play a critical role in breaking down complex carbohydrates such as starch, without which they cannot be absorbed because polysaccharides need to be broken down first into monosaccharides for absorption to take place [30]. The association between α-amylase inhibition and its potential contribution to the management of T2DM with phenolic extracts have been investigated for other vegetables [31,32].

α-glucosidase is another enzyme that makes glucose available in the body by breaking down oligo- and disaccharides, converting them into absorbable monosaccharides. Therefore, in order to reduce serum glucose levels and manage related diseases, α-glucosidase inhibition has been proposed as a suitable approach [33,34]. This work showed that the vegetable extracts had weaker α-glucosidase inhibition than acarbose, the drug. In fact, only brinjal extract at 8 and 10 mg/mL produced similar inhibitory values to acarbose at 0.25 mg/mL. In contrast, a nanoparticulated ethanolic extract of bitter gourd was reported to have stronger α-glucosidase inhibition than acarbose [29]. Nwanna, Ibukun, and Oboh [35] reported 50% inhibition of α-glucosidase activity for a similar eggplant variety using 63.24 ± 0.30 µg/mL, which is stronger than the results obtained in this work. The difference may be due to their use of solvent extraction, which could have isolated more compounds than the aqueous extraction used in the present work. The observed variations in identifiable polyphenolic compounds of the vegetable extracts did not have strong effects in influencing α-glucosidase inhibition. However, the results obtained in this work agree with a previous work [30], which suggested that brinjal phenolics may have potential for use in controlling T2DM because of the strong glucosidase-inhibitory property. A previous work [36] reported 66.64% (at 2.5 mg/mL), which is higher than the 48.65% (at 6 mg/mL) obtained in this work. The α-glucosidase inhibitory properties of a sample have been shown to be dependent on the type of extracting solvent; for example, ethyl acetate extract had stronger activity than hexane, methanol, and chloroform extracts of bitter gourd [36]. Therefore, the weaker activity obtained for the bitter gourd extract in this work may be due to the aqueous extraction as compared to the ethyl acetate extract.

Lipase is secreted in the pancreas and is used as a catalyst to aid triglyceride hydrolysis in the stomach, and this hydrolysis is completed by intestinal lipase in the small intestine [28,37]. It has been estimated that about 50–70% of total dietary fats are hydrolyzed for absorption by PL [38], which emphasizes the importance of this enzyme in calorie release from diets. Surprisingly, orlistat is the only FDA-approved drug for inhibiting PL. It was reported that orlistat can prevent approximately 30% of dietary fat absorption; however, regular usage is associated with some undesirable side effects like flatulence, diarrhea, oily spotting, incontinence, abdominal cramping, and fecal urgency [39]. Therefore, the search for active PL inhibitors, especially natural compounds with potentially fewer side effects, has become important. Results from this work confirmed the PL-inhibitory activity of the vegetable extracts, especially the stronger effect of Indian spinach as compared to orlistat. Therefore, the Indian spinach extract could serve as a potential agent for use in limiting PL-dependent digestion of dietary lipids. While there may be contributions from other polyphenolic compounds, the results suggest that the unique combination of vitexin with the kaempferol and vitexin glycosides contributed to the stronger PL inhibition by Indian spinach. This is because ash gourd and snake gourd, which contain vitexin and kaempferol glycosides, respectively, but not both types of polyphenols, were not as active as Indian spinach. The activities obtained in this work are weaker than the 92.0 ± 6.3 to 128.5 ± 7.4 μg/mL reported for the polyphenolic-rich extracts of common bean varieties [10]. The differences may be due to the type of sample and the extraction solvent since acetone was used as compared to aqueous extraction in the present work.

ACE is responsible for the formation of angiotensin II, a powerful vasoconstrictor; excessive physiological levels of angiotensin II lead to hypertension [15]. Therefore, ACE activity inhibition has been used to prevent and treat various diseases like heart failure, myocardial infarction, nephropathy, and even diabetes. However, apart from the different negative side effects associated with the use of pharmacological agents, ACE-inhibitory drugs are not permitted for use during pregnancy due to potential damage to the fetus [40]. Therefore, foods may serve as sources of ACE-inhibitory compounds with less harmful effects. For example, flavanols have been shown to inhibit in vitro activity of ACE as well as in isolated rat kidney membranes [41]. The vegetable extracts had lower ACE inhibition than captopril, which is consistent with the strong antihypertensive activity associated with standard drugs. However, the advantage of natural products is the reduced risk of negative side effects that could make them preferred antihypertensive agents when compared to synthetic drugs. Moreover, it is possible to incorporate these vegetable extracts into foods for regular consumption to combat the incidence of hypertension [21]. The results suggest that the presence of multiple compounds as identified in this work produced no strong synergistic effect since the strongest ACE inhibition was associated with samples where one polyphenolic compound predominate. However, it should be emphasized that other non-identified compounds may have contributed to the observed activities. The results are consistent with previous reports, which have reported that ACE-inhibitory activity varies because of differences in polyphenolic compounds of different plant extracts [21,42,43,44,45].

Renin inhibition has also been shown to be an effective means of reducing blood pressure because it catalyzes the rate-determining step (conversion of angiotensinogen to angiotensin I) in the RAS pathway [15,46]. However, effective renin-inhibitory drugs are rare and only one (aliskiren) has so far been approved as an antihypertensive agent [47]. Therefore, natural compounds that inhibit renin activity could enhance the management of hypertension. This is possible because polyphenolic compounds such as saponins have been shown to bind renin protein [48] and produce blood pressure-lowering effects after oral administration to rats [49]. As shown in Figure 5, the vegetable extracts had weaker renin inhibition than aliskiren even though the drug was used at 10 times lower the concentration of the extracts. The weaker renin inhibition by the vegetable extracts is consistent with the high purity and chemical potency of the synthesized drug. However, brinjal extract had the strongest renin inhibition among the vegetables, which suggests that this sample could serve as a potential natural product to reduce renin activity. Unlike ACE inhibition, the presence of multiple polyphenolic compounds may have worked synergistically to contribute to the renin-inhibitory activity of Indian spinach, kangkong, and okra. Reports of polyphenol-dependent inhibition of renin activity are scant, but a previous work with aqueous vegetable leaf extracts reported <40% inhibitions, although at a lower concentration of 0.25 mg/mL [22]. Strong inhibition of in vitro renin activity was also reported for purified soybean saponins with an IC_50_ value of 59.9 µg/mL [49]. The high purity of the saponin preparation could have contributed to the high renin inhibition when compared to the mixture of polyphenolic compounds used in this work.

## 5. Conclusions

Results from this work have shown that the aqueous extracts of vegetables could inhibit enzyme activities with respect to obesity, diabetes, and hypertension. The extracts were more effective inhibitors of α-amylase than α-glucosidase, which suggests a strong potential to limit excessive glucose release during digestion because starch hydrolysis is the rate-limiting step. Ability of the extracts to also inhibit PL suggests potential use as agents to limit the release of fatty acids during intestinal digestion, which could assist in reducing hyperlipidemia. Inhibition of ACE and renin activities are indications of potential blood pressure-reducing ability, which could also make the extracts function as antihypertensive agents. Overall, Indian spinach and brinjal extracts produced the most promising inhibitory effects on the five enzymes studied in this work, whereas stem amaranth extract was the poorest. However, in vivo determination of enzyme inhibitory activities using suitable animal disease models is required to confirm potential health benefits.

## Figures and Tables

**Table 1:** Main polyphenolic compounds of aqueous extracts of vegetables *.

Samples	Polyphenolic Compounds	Retention	*m/z*	MS/MS	Concentration	
		Time (min)	(Da)	(Da)	(mg/g)	
					A ^1^	B ^2^
Ash Gourd	Vitexin ramnoside	3.2	579	433, 313, 283	1.23	0.77
Brinjal	Chlorogenic acid	2.4	355	163	5.63	4.07
Indian spinach	Vitexin arabinoside	3.0	465	433, 313, 283	8.96	3.40
	Vitexin	3.2	433	313, 283	2.82	1.07
	Kaempferol O-rutinoside	3.4	595	449, 287	0.12	0.05
Kangkong	Dicaffeoyl quinic acid	3.5	517	163	6.92	3.72
	Quercetin hexoside	3.4	465	163	4.46	2.40
	Chlorogenic, isochlorogenic acid	2.5	355	163	10.12	5.43
Okra	Quercetin O-sophoroside	3.0	627	301	3.33	2.28
	Quercetin O-hexoside	3.4	465	301	2.63	1.80
	Quercetin malonyl O-hexoside	3.5	551	301	0.66	0.45
Snake gourd	Kaempferol O-sophoroside	3.1	611	287	81.35	57.15

* Compounds in bitter gourd, ridge gourd, and stem amaranth could not be identified. ^1^ Dried polyphenolic extract. ^2^ Dried vegetable powder.
